# An international road map to improve pain assessment in people with impaired cognition: the development of the Pain Assessment in Impaired Cognition (PAIC) meta-tool

**DOI:** 10.1186/s12883-014-0229-5

**Published:** 2014-12-10

**Authors:** Anne Corbett, Wilco Achterberg, Bettina Husebo, Frank Lobbezoo, Henrica de Vet, Miriam Kunz, Liv Strand, Marios Constantinou, Catalina Tudose, Judith Kappesser, Margot de Waal, Stefan Lautenbacher, on behalf of EU-COST action td 1005 ‘Pain Assessment in Patients with Impaired Cognition, especially Dementia’ Collaborators: http://www.cost-td1005.net/

**Affiliations:** Wolfson Centre for Age-Related Diseases, Kings College London, London, UK; Department of Public Health and Primary care medicine, Leiden University Medical Center & EU COST Vice-Chair, Leiden, The Netherlands; Department of Global Public Health and Primary Care, Centre for Elderly and Nursing Home Medicine, University of Bergen, Bergen, Norway; Center for Age-Related Medicine, Stavanger University Hospital, Stavanger, Norway; Department of Orofacial Pain and Dysfunction, Academic Centre for Dentistry Amsterdam (ACTA), University of Amsterdam and VU University Amsterdam, Amsterdam, The Netherlands; Department of Epidemiology & Biostatistics, EMGO Institute for Health and Care Research, VU University medical center, Amsterdam, The Netherlands; Department of Physiological Psychology, University of Bamberg, Bamberg, Germany; Department of Global Public Health and Primary Care, Physiotherapy Research Group, University of Bergen, Bergen, Norway; Social Sciences Department & Center for Cognitive and Behavioral Psychology (CCBP), University of Nicosia, Nicosia, Cyprus; Department of Neurology, Neurosurgery and Psychiatry, University of Medicine and Pharmacy “Carol Davila”, Bucharest, Romania; Department of Clinical Psychology, University of Giessen, Giessen, Germany; Department of Public Health and Primary Care, Leiden University Medical Center, Leiden, The Netherlands; Department of Physiological Psychology, University of Bamberg, Bamberg, Germany

**Keywords:** Pain, Dementia, Assessment, Tool, EU-COST, Cognition

## Abstract

**Background:**

Pain is common in people with dementia, yet identification is challenging. A number of pain assessment tools exist, utilizing observation of pain-related behaviours, vocalizations and facial expressions. Whilst they have been developed robustly, these often lack sufficient evidence of psychometric properties, like reliability, face and construct validity, responsiveness and usability, and are not internationally implemented. The EU-COST initiative “*Pain in impaired cognition, especially dementia*” aims to combine the expertise of clinicians and researchers to address this important issue by building on previous research in the area, identifying existing pain assessment tools for dementia, and developing consensus for items for a new universal meta-tool for use in research and clinical settings. This paper reports on the initial phase of this collaboration task.

**Methods:**

All existing observational pain behaviour tools were identified and elements categorised using a three-step reduction process. Selection and refinement of items for the draft Pain Assessment in Impaired Cognition (PAIC) meta-tool was achieved through scrutiny of the evidence, consensus of expert opinion, frequency of use and alignment with the American Geriatric Society guidelines. The main aim of this process was to identify key items with potential empirical, rather than theoretical value to take forward for testing.

**Results:**

12 eligible assessment tools were identified, and pain items categorised according to behaviour, facial expression and vocalisation according to the AGS guidelines (Domains 1 – 3). This has been refined to create the PAIC meta-tool for validation and further refinement. A decision was made to create a supporting comprehensive toolkit to support the core assessment tool to provide additional resources for the assessment of overlapping symptoms in dementia, including AGS domains four to six, identification of specific types of pain and assessment of duration and location of pain.

**Conclusions:**

This multidisciplinary, cross-cultural initiative has created a draft meta-tool for capturing pain behaviour to be used across languages and culture, based on the most promising items used in existing tools. The draft PAIC meta-tool will now be taken forward for evaluation according to COSMIN guidelines and the EU-COST protocol in order to exclude invalid items, refine included items and optimise the meta-tool.

**Electronic supplementary material:**

The online version of this article (doi:10.1186/s12883-014-0229-5) contains supplementary material, which is available to authorized users.

## Background

There are an estimated 35.6 million people with dementia worldwide and this figure continues to rise [1]. The condition represents a significant public health issue, yet treatment and care often fall below basic standards due to the unique and complex challenges presented by dementia. Identification and assessment of pain in people with dementia present a particular challenge. It is thought that up to 80% of people with dementia living in care homes regularly experience pain [2]. However, there is conflicting evidence regarding the incidence of pain in people with dementia. A number of studies have reported lower prevalence of pain in these individuals compared to healthy older adults, although it is also likely that pain is under-reported due to the difficulties experienced by health care professionals in detecting it [3,4]. There is some limited evidence for altered pain pathways as a result of the pathology associated with dementia which includes degeneration of pain centres in the medial pain system [5,6]. Some studies have also reported an increased threshold of pain tolerance in people with Alzheimer’s disease (AD) [7]. However this evidence is conflicting, with other studies reporting no alteration or increases in pain processing in AD as measured through EEG, fMRI, psychophysical and observational measures [8]. As yet the detection of pain through these approaches is imperfect and this should be taken into account when interpreting these studies.

Pain in dementia is usually related to musculoskeletal, gastro-intestinal and cardiac conditions, genito-urinary infections, pressure ulcers and oral pain. Neuropathic pain, defined as pain caused by a lesion or dysfunction in the central nervous system, is common in dementia. This is particularly the case in people with vascular dementia (VaD) due to the high prevalence of diabetes, and stroke in this group, leading to deafferentiation, a form of central neuropathic pain caused by white matter lesions [9,10]. Approximately 35% of stroke patients are known to suffer from post-stroke central neuropathic pain [11]. Furthermore, Scherder et al. recently suggested that central neuropathic pain is by far the most undertreated type of pain in patients with dementia [12]. Despite these numerous established causes of pain, international epidemiological research has reported that the use of pain medication is often inappropriate in this patient group [7]. This is particularly prominent in care home and hospital settings where people are likely to have more severe cognitive impairment and are reliant on prescription of analgesics by health professionals. A large number of studies have emphasised the challenge of assessing pain in people with dementia in these settings, and it is likely that this is the primary contributing factor to under-treatment of pain in these individuals [7,13].

Thorough assessment of pain is essential to ensure effective treatment and ongoing care. In most patient groups the most effective method of identifying pain is through self-report. However, a key symptom of dementia is the loss of ability to communicate, particularly in the later stages of the condition. In addition, these people often lack insight into their condition. These factors combined mean that people with dementia do not have the ability to give an accurate report of their pain, its duration, location and severity. As a result the majority of general pain assessment tools are often partially inappropriate for use in dementia. A key element of any assessment tool for dementia would be the observation of pain-related behaviours as substitutes for verbal reports of pain, especially in moderate and severe dementia, to detect the presence and intensity of pain by a trained observer. In addition, in order to provide a full picture of the pain an ideal tool would also enable assessment of the location and duration of pain through extended observation of the individual. Differentiation of pain states varies in complexity. Whilst spontaneous, acute pain, for example after a fall, is relatively easy to identify through the resulting facial, verbal, and behavioural pain indicators, chronic pain is more difficult to detect, requiring identification of more indirect pain avoidance behaviours such as reduced movement or apathy. Musculoskeletal pain can often be identified through monitoring responses to guided movements whilst visceral and neuropathic pains are more challenging to detect [14]. Oral pain is also particularly dominant in this patient group, yet no existing pain assessment tools include oral pain as a key factor. A recent review identified this need, and highlighted the inclusion of pain-related items such as modified oral movement and behaviours as key to a comprehensive tool [15]. An ideal pain assessment tool would capture a combination of all these aspects to provide a broad view of the pain experienced by an individual at one point in time. It should be noted however that the subjective experience of pain and the complexity of the issue, particularly in a patient group where comorbidities and communication impairments are common, makes the development of such a perfect tool extremely unlikely. Therefore, the most important objective of a pain assessment tool must be to enable detection of pain and its approximate intensity to enable further examination and appropriate treatment and to monitor the effects of treatment.

In 2002 the American Geriatric Society (AGS) published guidance which provides a useful framework for developing an assessment tool for pain in dementia (Table 1) [16]. A number of observational and informant-based assessment tools have been developed based on identification of specific behaviours, many of which align closely with the AGS guidelines. The tools are applied by a proxy rater, usually a caregiver (health professional or family carer) who is familiar with the individual, and combine observation of behaviours, emotions, interactions and facial expressions. A number of systematic reviews have examined the range of tools currently available for use in dementia. One recent review concludes that there are 12 promising pain assessment tools instruments available, but the majority of these require further validation in people with dementia and for day-to-day use in clinical settings [7]. Whilst many of the available tools have been developed through robust methodology, including intensive observation in the clinic, consultation with users and patients, and refinement of items, the existing tools are disparate, with no one universal tool. In particular, whilst there is some agreement between existing tools on the concepts for pain assessment, there is great disparity in the methods by which they are operationalized. Importantly, existing tools frequently lack comprehensive data on face and construct validity, reliability and responsiveness. Few dictate the specific situation in which assessment should take place, for example during rest, guided movement or during daily activities, nor have the majority been developed for ease of use in clinical settings and clinical utility. As a result, no truly universal tool for detection of pain in dementia exists. There remains an urgent need to draw on the currently available resources and to develop an easy-to-use assessment tool which has utility in both research and clinical settings, and robust validation data to support its implementation.

**Table 1:** **Common Pain Behaviours in Cognitively Impaired Elderly Persons according to the AGS Panel on Persistent Pain in Older Persons** [16]

**Domain**	**Items**
**1: Facial expressions**	Slight frown/sad or frightened expression
	Grimacing, wrinkled forehead, closed or tightened eyes
	Any distorted expression
	Rapid blinking
**2: Verbalisations & vocalisations**	Sighing, moaning, groaning
	Grunting, chanting, calling out
	Noisy breathing
	Asking for help
	Verbally abusive
**3: Body movements**	Rigid, tense body posture, guarding
	Fidgeting
	Increased pacing, rocking
	Restricted movement
	Gait or mobility changes
**4: Changes in interpersonal interactions**	Aggressive, combative, resisting care
	Decreased social interactions
	Socially inappropriate, disruptive
	Withdrawn
**5: Changes in activity patterns or routines**	Refusing food, appetite change
	Increase in rest periods
	Sleep, rest pattern changes
	Sudden cessation of common routines
	Increased wandering
**6 Mental status changes**	Crying or tears
	Increased confusion
	Irritability or distress

A major problem in the development of pain assessment tools is that the scope of distinct pain-related behaviours is difficult to distinguish from other behavioural symptoms that commonly arise in people with dementia. These individuals frequently develop symptoms such as agitation, aggression and apathy, collectively described as the Behavioural and Psychological Symptoms of Dementia (BPSD). 90% of people with dementia experience BPSD at some point in their condition, and this is most common in the more severe stages of their impairment. The underlying causes of BPSD are varied and complex. Pain is a major contributor, and any comprehensive pain assessment tool must consider and include BPSD to ensure these factors are identified and included in a diagnosis. There is emerging evidence indicating that verbal agitation behaviours such as complaining, negativism, repetitious sentences and questions, constant request for attention, and cursing or verbal aggression respond to pain treatment. In addition, certain BPSD such as restlessness and pacing have been shown to be sensitive to treatment with analgesics [17]. However, the definition of true pain-related behaviours is vague, and varies widely between individuals. This complication is likely to lead to incorrect detection of pain or and inaccurate diagnosis of BPSD due to the underlying causative pain. This inaccuracy in diagnosis can result in inappropriate treatment, including the use of antipsychotic medications to address BPSD unnecessarily. Conversely, people may be treated with analgesics under the assumption of underlying pain in the absence of a meaningful assessment, and receive analgesia without due cause. A valid pain assessment tool must therefore consider the importance of both specific and non-specific pain indicators to enable first detection of pain and then differential diagnosis of the underlying cause in order to support appropriate treatment and care.

The EU-COST action “Pain in impaired cognition, especially dementia” was initiated in 2011 and currently includes representatives from 16 European nations. It is a four-year initiative which aims to draw on the combined clinical, research and methodological expertise of its members which include nurses, geriatricians, psychiatrists, anaesthesiologists, neuropsychologists, psychotherapists, physiotherapists, palliative care experts and experts in clinimetrics, in addition to physiological/experimental (human and animal) researchers. The group also includes a number of authors of previous pain assessment tools. The COST action is coordinated by a central Management Committee. Five Working Groups (Psychometrics and Algesimetry, Nursing and Care, Clinical Evaluation and Epidemiology, Experimental Evaluation and Palliative Care) have been created to oversee specific areas, all of which are integrated with representative members and cross-working to ensure effective collaboration. The primary aim of the collaboration is to establish the current situation of pain assessment in dementia and to develop and evaluate a comprehensive international tool for use in both clinical and research settings. Key elements of the proposed tool are summarised in Table 2. The multidisciplinary, cross-cultural approach will allow for a novel ‘joined-up’ approach to development and evaluation, incorporating international contributions and expertise throughout the process from all key stakeholder groups. The decision to create a meta-tool based on existing instruments was informed by a thorough review of the literature and current clinical practice which revealed the absence of a single tool for use in all settings which is embedded in the practicalities of clinical practice and user-based design. The initiative aims to develop a truly unique meta-tool which, instead of being developed from patient observations, is based on the scrutiny and inclusion of items from existing assessment tools based on empirical evaluation of each item. This innovative approach ensures the best, most informative are used. Furthermore, the meta-tool will form part of a more comprehensive toolkit which will provide supporting resources and guidance to capture the nuances of pain in dementia including the specific needs of assessing pain in different locations and settings, and to support decision-making regarding the most suitable treatment. This will also support assessment in distinct patient groups including Parkinson’s Disease Dementia, Huntington’s disease and delirium. Thorough evaluation of the meta-tool and the associated toolkit will conform to the newest international criteria for development and testing of measurement instruments, as have been described by the international COSMIN group [18].

**Table 2:** **Key elements of the proposed PAIC tool and toolkit**

**Theme**	**Required element**
**Process**	• Makes use of the best items from existing instruments, developing a meta-tool with a pool of useful items
	• To provide a toolkit, not a single tool, out of which instruments can be created for different contexts of application (type of cognitive impairment, setting etc.)
	• Provide potential to create specific tools (or additional scales) for specific pain associated conditions (such as oral-facial pain, back pain, neuropathic pain)
	• Includes a guideline-and web-based, multilingual application
	• Instruments for use in both clinical practice and research
	• Creation and validation of instruments follows a predefined process, following the COSMIN* criteria.
**Quality**	• Practicable and feasible in different settings (home care, long term care, palliative care and acute hospital care) and different countries (with first the focus on the Western world)
	• Sound psychometric properties, i.e. a reliable and valid instrument
	• Sensitive to change, i.e. identify new pain and detect changes after successful intervention, for instance with pain medication
	• Feasible and valid in several important groups of people with cognitive impairment, such as dementias, coma/pvs and people with a mental handicap or learning disability.

*COSMIN: COnsensus-based Standards for the selection of health Measurement [18].

This paper describes the first phase of the EU-COST action programme of research which incorporates a thorough review of existing assessment tools for pain in dementia and the systematic evaluation of individual elements to create an initial draft meta-tool for further evaluation.

## Methods

### Search strategy

A systematic search of the PubMed (N = 186), EMBASE (N = 143) and Cochrane (N = 11) databases was conducted to identify reviews of pain assessment tools published between 2005 and 2011. An updated search was conducted in September 2012 using the same criteria. The search was conducted in collaboration with the library at the University of Bergen, Norway. The following search terms were used: “dementia” OR “Alzheimer`s disease”, AND “pain”, “aggression”, “neuropsychiatric”, “pain prevalence”, “pain diagnoses”, “pain assessment”, “pain assessment instruments”, “pain assessment recommendation”, “pain behaviour”, “management”, “treatment”, “analgesics” and the names of individual and non-pharmacological treatments AND “nursing home”, “treatment recommendation”, “review”, “randomized clinical trial”.

### Inclusion of pain assessment tools

Pain assessment tools included in this review had been published as measures and recommended in review articles between 2005 and 2011. The tools were available online or through a published article, and had been reported as useful in clinical practice for patients with dementia. Priority was placed on tools with available published data for reliability, face validity and sensitivity to change, although such evidence was limited. Furthermore, tools were selected with some indication of feasibility for use in various settings (home care, long term care, palliative care and acute hospital care) and different countries, with first the focus on the Western world, sound psychometric properties, and good psychometric properties in more than one group of people with dementia, including different types of dementia, coma or people with a learning disability. Flexibility in eligibility criteria was allowed to account for the different levels of supporting evidence. Fulfilment of eligibility criteria was confirmed through an expert consensus process including an expert panel consisting of members of the Working Groups in the EU-COST action. This rigorous selection approach ensured that the process resulted in the best possible pool of pain assessment items with which to create the new tool.

### Expert panel

The review and refinement strategy was led by a series of expert panels unique to COST Action TD 1005. Psychometric scale development was primarily performed by the Psychometrics and Algesimetry Panel, which had met 11 times since the start of the Action in 2011. The panel consisted of a multidisciplinary team with expertise in pain, dementia, psychology, nursing, geriatric medicine and clinimetrics who contributed to a series of consensus meetings conducted in person. This panel was constantly interacting with other panels responsible for usability, clinical and experimental validation and reliability testing and consideration of special situations like end of life care. This approach focused on theoretically and empirically extracting and refining pain assessment items to develop a meta-tool based on the evidence base.

### Review strategy

The panel scrutinised all included tools. Each assessment item was pooled to create an assessment database. This followed a three step reduction process. First, each item was allocated to the six domains published by the AGS [16]and the frequency with which each individual assessment item appeared in the total pool was recorded to provide an indication of the representation of each item in existing validated scales. Items with a frequency of two and more automatically qualified for further consideration. Items with a frequency of less than two were included if they were derived from an instrument with robust published evidence of the instrument’s development and validation (such as ‘empty gaze’ from DOLOPLUS2). This process informed the subsequent consensus process to ensure consideration of the relative importance and use of specific item types. Second, the item pool was refined to remove repetitions. Finally, items were further categorised according to expert consensus within three main categories of the AGS guidelines – facial expression, vocalisation, and body movements.

These three AGS guideline domains were selected for further scrutiny by expert panel consensus as they were considered to be the most relevant, building on previous research regarding pain behaviour, and aspects covered by other assessment tools. The remaining three domains (interpersonal interactions, changes in patterns or routines and change in mental status) were excluded from the main tool for two key reasons. Firstly, a number of the items from domains four, five and six that were considered most relevant to cognitive impairment, such as aggression, resistance to care and confusion were already represented in domains one, two and three. An example of this was restlessness, which fulfilled both categories of body movement (AGS Domain 3) and changes in activity patterns or routines (AGS Domain 5). Secondly, it was felt that a number of pain-related behaviours within the excluded domains were more broadly symptomatic of dementia and so might unnecessarily reduce the specificity of the tool itself. For example, changes in appetite and insomnia commonly arise as part of the progression of dementia and are not specific to pain and AGS Domains one, two and three. Items within the fourth, fifth and sixth AGS domains were therefore excluded from the main meta-tool, but planned for inclusion within the future overall toolkit through existing measures, for example to assess BPSD and mood.

### Identification of candidate pain assessment items for the PAIC tool

The full membership of the COST Action TD 1005 expert panels oversaw the selection of pain assessment items for inclusion in the preliminary draft of the Pain Assessment in Impaired Cognition (PAIC) tool, under the governance of the Psychometrics and Algesimetry panel. First, each panel was consulted on the output of the review strategy described above to form a second consensus and refinement process. Informed recommendations collated by the chair of each panel resulted in the creation of subcategories within each category described above to ensure that they covered a broad perspective within each. At least one item from each sub-category was included in the draft PAIC tool.These subcategories provided structure for the item reduction process to ensure critical areas were not excluded and to provide an accurate view of the coverage and breadth of the items as they were refined. This process also strengthened the empirical approach taken in this study. Decisions to include items were guided by published evidence of use and indication of ability to capture pain in addition to the panel’s expert opinion regarding their clinical utility and accuracy in pain detection. Scoring systems utilised in each tool were recorded and evaluated. The combined expertise of the panel informed the identification of systems that best balanced usability with psychometric precision. Clear definitions of each item and instructions for use were created by the expert panel and preserved for accuracy through consultation with native English speakers.

## Results

### Identification of pain assessment tools

Nine reviews of pain assessment tools were identified [15,19-26]. A further two reviews were identified in the updated search [7,13]. Scrutiny of these reviews identified 12 tools fulfilling the eligibility criteria which were also agreed through expert consensus with the Working Groups in the EU-COST action. The included assessment tools were the ABBEY Pain Scale [27], ADD [28,29], CNPI [30,31], DS-DAT [32,33], DOLOPLUS-2 [34], EPCA-2 [35], MOBID-2 Pain Scale [14,36], NOPPAIN [37], PACSLAC [38], PAINAD [39], PADE [40], and PAINE [41] (Table 3, see Additional file 1). Following scrutiny of the available validation data and discussion of clinical utility the panel agreed that no one tool showed the required feasibility and level of evidence of reliability, validity and responsiveness or showed full, appropriate clinical utility.

**Table 3:** **Summary of evaluation of included pain assessment scales in published systematic reviews**

	**Stolee et al.** [24]	**Zwakhalen et al.** [26]	**Herr et al.** [21]	**Hadjistavropoulos et al.** [20]	**van Herk et al.** [25]	**Chapman et al.** [19]	**Park et al.** [23]	**Lobbezoo et al.** [15]	**Herr et al.** [22]	**Corbett et al.** [7]	**Husebo et al.** [13]
**No of scales included in reviews**	10	12	10	11	13	3	11	9	15	12	12
**Abbey** [27]		x	x	x	x		x		x	x	X
**ADD** [28,29]			x		x		x	x		*X*	*X*
**CNPI** [30,31]	x	x	x	x	x		x		x	x	X
**DS-DAT** [32,33]	x		*X*	X	*X*		*X*	x	x	x	X
**DOLOPLUS 2** [34]	x	*X*	x	*X*	x			x	x	x	X
**EPCA** [35]	x	x				*X*			x	x	X
**MOBID-2** [14,36]										*X*	*X*
**NOPPAIN** [37]		x	*X*	*X*	x		x		x	x	X
**PACSLAC** [38]		*X*	x	*X*	*X*		*X*	x	*X*	x	X
**PAINAD** [39]	x	x	x	x	*X*		x	x	*X*	x	X
**PADE** [40]	x	x	x	*X*	x		x		x	x	X
**PAINE** [41]									x	x	X

'x' indicates scale included in the published systematic review; '*X*' indicates scales recommended by the review.

### Categorisation and refinement of assessment items

Item identification, frequency scoring and categorization were completed for all included scales. Expert consensus was reached regarding item selection. The panel agreed that items within AGS domains 1–3 (Facial expression, Vocalization and Body movements) were the most promising for further exploration. The panel agreed to explore the use of existing validated tools for assessment of AGS domains four, five and six, which will form part of the future toolkit. The facial expression category was divided into the subcategories cognition, emotion, anatomical orientation, autonomic reaction and qualitative judgment of expression. For example, the cognition sub-category was created to define the items ‘empty gaze’ and ‘seeming disinterested’, which appeared sufficiently often as indicative pain behavior in the included scales, and were agreed within the expert panel to reflect cognitive impairment. The vocalization category consisted of verbal utterances, nonverbal utterances and breaths, and the body movement category was split into tension, defense, pain relieving adjustments and restlessness (Table 4). Repeated items, or items that overlapped to a large extent, were removed. A high degree of consistency and repetition was recorded for each scale. The highest rate of consistency was within facial expressions items where 41% of items were found in at least two scales.

**Table 4:** **Categories of items identified by the expert panel in domains 1–3 in the American Geriatric Society guidance**

**Facial Expression (AGS domain 1)**	**Vocalization (AGS domain 2)**	**Body movement (AGS domain 3)**
1. Cognition	1. Verbal utterances	1. Tension
2. Emotional state	2. Nonverbal utterances	2. Defensive behaviour
3. Anatomically-based descriptions	3. Breath	3. Pain relieving adjustments
4. Autonomic reactions		4. Restlessness
5. Qualitative judgment of expression		

### Selection of items for inclusion in the PAIC tool

For each of the 12 sub-categories within the domains of Facial Expression, Body movements and Vocalisation, the expert panel selected the most promising pain assessment items within each sub-category. Selection was based on frequency of use of items in published assessment scale (Table 5). Further selection was then based on interpretation by the expert panel, informed by published laboratory and clinical observation and experimental studies, documented response to pain treatment through existing studies including clinical trials and clinical experience of the panel.

**Table 5:** **Rationale for inclusion of pain assessment items in PAIC**

	**Frequency of use in published tools**	**Item explanation derived from published tools**
**FACIAL EXPRESSION**		
**Pained expression**	1	Facial display of pain
**Frowning**	7	Lowering and drawing brows together
**Narrowing eyes**	1	Grimacing, narrowed eyes with tension around the eyes
**Closing eyes**	3	Not just blinking
**Raising upper lip**	1	Grimacing, upper lip raised, nose may be wrinkled
**Opened mouth**	1	The lips are parted, jaw is dropped
**Tightened lips**	2	Lips are pressed together and appear more narrow
**Clenched teeth**	3	Teeth are pressed together with tension
**Empty gaze**	1	Eyes do not reflect any emotion or thinking activity (“blank expression”)
**Seeming disinterested**	1	Face does not reflect any interest in the environment
**Pale face**	1	Pale skin colour
**Teary eyed**	5	Watery eyes
**Looking tense**	3	Facial display of strain or worry
**Looking sad**	6	Facial display of unhappiness, sorrow or low mood
**Looking frightened**	6	Facial display of fear, alarm or heightened anxiety
**BODY MOVEMENTS**		
**Freezing**	2	Tense, sudden stiffening, rigid, avoiding movement, holding breath
**Curling up**	3	Curling up the body tightly, pulling in arms and legs
**Clenching hands**	4	Tensing hands, making fists, grabbing objects tightly
**Resisting care**	5	Resisting being moved or resisting care, being uncooperative
**Pushing**	2	Actively pushing somebody or something away
**Guarding**	7	Protecting affected area, holding body part, avoiding touch, moving away
**Rubbing**	3	Tugging or massaging affected area
**Limping**	3	Avoiding pain while walking in an uneven way
**Restlessness**	9	Fidgeting, agitation, rocking back and forth
**Pacing**	2	Wandering restlessly back and forth (might also be in a wheelchair)
**VOCALISATION**		
**Using offensive words**	2	Cursing, swearing, or using foul language
**Using pain-related words**	5	Using pain words, like “ouch”, “ow”, or “that hurts
**Repeating words**	2	Repeating words or phrases again and again (not stuttering)
**Complaining**	3	Expressing being unhappy, sick, uncomfortable, and/or in pain
**Shouting**	1	Using a loud voice to express words
**Mumbling**	3	Uttering words and/or sounds indistinctly
**Screaming**	4	Using a loud and/or high-pitched voice to express sounds
**Groaning**	10	Moaning, making a deep, inarticulate sound
**Crying**	10	Whimpering, sobbing, wailing, or weeping
**Gasping**	4	Breathing sharply, laboriously, and/or loudly
**Sighing**	3	Taking in and letting out a long, loud breath

This process resulted in a final item pool for each of the 12 subcategories within the three domains to be taken forward for reliability and validity testing in clinical and experimental settings. A number of items included in the tool were considered by the panel to be potentially less accessible to an observer than others, and more dependent on unsystematic inference. The decision was made to include these items in the draft tool to enable further empirical item reduction during the validity and reliability testing so as to avoid making a-priori assumptions on the utility of these elements of the tool.

### Creation of preliminary draft of the PAIC tool for experimental and clinical testing

The expert panel came to consensus regarding the format and scoring structure for each assessment item. Key factors were ease of use and clarity for the user regarding the meaning of each score. It was agreed that at this preliminary stage a graded scoring system would be taken forward for testing. Based on the literature, a four-point Likert scale was developed to enable rating of each item [42,43]. It was agreed that this would produce a sensitive measure of presence of pain, in addition to providing a measure of pain intensity. This was considered to be critical for use in research settings in order to assess the impact of an intervention on pain. It is therefore expected that this format would be used in research settings. The panel emphasised that for use in clinical practice a dichotomous response (present/absent) may support ease of use, particularly in settings such as care homes where simplicity and usability is a key consideration in implementation. It is expected this simpler format will be produced following the full evaluation of the scale prior to dissemination and this will be a focus of future development. It was recognised that the refinement needed to create a simple clinical tool may be challenging, particularly due to the need to retain sensitivity to change which may be lost if the four-point scale is removed.

Definitive descriptions of each item and instructions for use of the tool were adapted from the original source tools through expert panel discussions and through consultation with a native English speaker. The final draft version of the PAIC tool for validity and reliability testing in clinical settings is shown in Tables 6, 7 and 8.

**Table 6:** Pain assessment by observer ratings in PAIC tool: Facial Expressions items*

**FACIAL EXPRESSIONS**	**MEANING OF ITEMS**	**Not at all**	**Slight degree**	**Moderate degree**	**Great degree**	**Not scored**
						**a = Item is not clear**
						**b = Situation is unsuitable**
						**c = Physical status of person not suitable for scoring**
						**d = Other**
**Pained expression** ^a^	Facial display of pain	**0**	**1**	**2**	**3**	a b c d
**Frowning** ^a^	Lowering and drawing brows together	**0**	**1**	**2**	**3**	a b c d
**Narrowing eyes** ^c^	Narrowed eyes with tension around the eyes	**0**	**1**	**2**	**3**	a b c d
**Closing eyes** ^c^	Not just blinking	**0**	**1**	**2**	**3**	a b c d
**Raising upper lip** ^c^	Upper lip raised, nose may be wrinkled	**0**	**1**	**2**	**3**	a b c d
**Opened mouth** ^c^	The lips are parted, jaw is dropped	**0**	**1**	**2**	**3**	a b c d
**Tightened lips** ^c^	Lips are pressed together and appear more narrow	**0**	**1**	**2**	**3**	a b c d
**Clenched teeth** ^c^	Teeth are pressed together with tension in the jaw	**0**	**1**	**2**	**3**	a b c d
**Empty gaze** ^b^	Eyes do not reflect any emotion or active thought (“blank expression”)	**0**	**1**	**2**	**3**	a b c d
**Seeming disinterested** ^b^	Face does not reflect any interest in the environment	**0**	**1**	**2**	**3**	a b c d
**Pale face** ^d^	Pale skin colour	**0**	**1**	**2**	**3**	a b c d
**Teary eyed** ^d^	Watery eyes	**0**	**1**	**2**	**3**	a b c d
**Looking tense** ^e^	Facial display of strain or worry	**0**	**1**	**2**	**3**	a b c d
**Looking sad** ^e^	Facial display of unhappiness, sorrow or low mood	**0**	**1**	**2**	**3**	a b c d
**Looking frightened** ^e^	Facial display of fear, alarm or heightened anxiety	**0**	**1**	**2**	**3**	a b c d

*Instructions to users: Please record the appearance of each facial expression described in the table below. Rate the intensity of the expression from ‘not at all’ to ‘a great degree’. If an item is not scored please indicate why not.

Subcategories: ^a^emotion, ^b^cognition, ^c^anatomical orientation, ^d^autonomic reaction, ^e^qualitative judgment of expression.

**Table 7:** Pain assessment by observer ratings in PAIC tool: Body Movement items*

**BODY MOVEMENTS**	**MEANING OF ITEMS**	**Not at all**	**Slight degree**	**Moderate degree**	**Great degree**	**Not scored**
						**a = Item is not clear**
						**b = Situation is unsuitable**
						**c = Physical status of person not suitable for scoring**
						**d = Other**
**Freezing** ^a^	Sudden stiffening, avoiding movement, holding breath	0	1	2	3	a b c d
**Curling up** ^a^	Curling up the body tightly, pulling in arms and legs	0	1	2	3	a b c d
**Clenching hands** ^a^	Tensing hands, making fists, grabbing objects tightly	0	1	2	3	a b c d
**Resisting care** ^b^	Resisting being moved or resisting care, being uncooperative	0	1	2	3	a b c d
**Pushing** ^b^	Actively pushing somebody or something away	0	1	2	3	a b c d
**Guarding** ^b^	Protecting affected area, holding body part, avoiding touch, moving away	0	1	2	3	a b c d
**Rubbing** ^c^	Tugging or massaging affected area	0	1	2	3	a b c d
**Limping** ^c^	Avoiding pain while walking in an uneven way	0	1	2	3	a b c d
**Restlessness** ^d^	Fidgeting, wringing hands, rocking back and forth	0	1	2	3	a b c d
**Pacing** ^d^	Wandering restlessly back and forth (might also be in a wheelchair)	0	1	2	3	a b c d

Please record the occurrence of each body movement described in the table below. Rate the intensity of the movement from ‘not at all’ to ‘a great degree’. If an item is not scored please indicate why not.

Subcategory: ^a^tension, ^b^defense, ^c^pain relieving adjustments and ^d^restlessness**.**

**Table 8:** Pain assessment by observer ratings in PAIC tool: Vocalisation items*

**VOCALIZATION**	**MEANING OF ITEMS**	**Not at all**	**Slight degree**	**Moderate degree**	**Great degree**	**Not scored**
						**a = Item is not clear**
						**b = Situation is unsuitable**
						**c = Physical status of person not suitable for scoring**
						**d = Other**
**Using offensive words** ^a^	Cursing, swearing, or using foul language	0	1	2	3	a b c d
**Using pain-related words** ^a^	Using pain words, like “ouch”, “ow”, or “that hurts”	0	1	2	3	a b c d
**Repeating words** ^a^	Repeating words or phrases again and again (not stuttering)	0	1	2	3	a b c d
**Complaining** ^a^	Expressing being unhappy, sick, uncomfortable, and/or in pain	0	1	2	3	a b c d
**Shouting** ^a^	Using a loud voice to express words	0	1	2	3	a b c d
**Mumbling** ^a^	Uttering words and/or sounds indistinctly	0	1	2	3	a b c d
**Screaming** ^b^	Using a loud and/or high-pitched voice to express sounds	0	1	2	3	a b c d
**Groaning** ^b^	Making a deep, inarticulate sound	0	1	2	3	a b c d
**Crying** ^b^	Whimpering, sobbing, wailing, or weeping	0	1	2	3	a b c d
**Gasping** ^c^	Breathing sharply, laboriously, and/or loudly	0	1	2	3	a b c d
**Sighing** ^c^	Taking in and letting out a long, loud breath	0	1	2	3	a b c d

*Instructions for users: Please record each vocalisation described in the table below. Rate the intensity of each item from ‘not at all’ to ‘a great degree’. If an item is not scored please indicate why not.

Subcategory: ^a^verbal utterances, ^b^nonverbal utterances and ^c^breaths.

## Discussion

Pain in dementia is a critical clinical issue which presents significant challenges for treatment and care. As the numbers of people with dementia increase alongside the ageing population, assessment and treatment of pain in this patient group will become an increasingly important and potentially costly consideration. The EU-COST initiative aims to address this issue by drawing on existing evidence and leading experts in clinical, professional and academic fields and by strengthening international collaboration. The initiative aims to provide an international roadmap to a better understanding of pain in people with cognitive impairment and ultimately to improve the assessment of pain across all treatment settings. This paper describes the first phase of the collaboration. The objective is to combine evidence from the literature and published pain assessment instruments with clinical and methodological expertise to reach consensus within Europe on a core assessment tool for pain in people with dementia.

A review of existing assessment tools revealed a number of potentially useful tools. No one tool showed the required feasibility and level of evidence of reliability, validity and responsiveness or showed full, appropriate clinical utility according to the consensus process, inclusion criteria and expert opinion of the COST Action panel (Table 3). However, many of the existing tools identified by the review were based on robust development work with some validation data, thus providing a valuable pool of items from which to build a composite meta-tool. This is strengthened by the emerging theme of consistently utilised items across many of the scales, particularly in facial expressions, which support the value of this approach. The collaboration has agreed on the urgent need for one universal user-led pain assessment tool for use with people with dementia in various clinical and care settings. A key element in the development of a new pain assessment tool for dementia was the need to build on and improve on the existing evidence base and available tools rather than to develop a tool from scratch, hereby creating a meta-tool. A further important aim was to create a meta-tool with international consensus from stakeholders across many European countries in an effort to ensure comprehensive implementation of the final product. This is a critical point as a universal assessment tool would enable standardisation of both clinical practice and assessment in research, neither of which are currently possible with the existing pain assessment tools. Creation of the meta-tool was achieved through a systematic scrutiny of current tools to identify the most reliable, frequently used items for pain assessment followed by close working with healthcare professionals to ensure clinical utility from an early stage. Importantly, each decision-making step within the development of this meta-tool has been informed by key considerations in pain assessment. Selection of items for inclusion focussed primarily on measurement of the intensity of pain, with plans to include additional methods for identifying pain location and duration in the future full toolkit. This approach, overseen by an experienced expert panel, has ensured the meta-tool includes the best items for detection of pain across the broad spectrum experienced by this patient group. The panel recognises the potential limitation to this methodology which centres around the assumption that the key concerns and challenges in assessment of pain in cognitive impairment have already been recognised. This raises the possibility that issues neglected by the literature or unrecognised within the panel may not be incorporated into the work. However, this risk has been mitigated by ensuring a robust methodology for the literature review and the broad cross-section of expertise and experience within the expert panel.

A critical aspect of this work is the unique approach taken that differentiates the PAIC meta-tool from approaches taken to develop previous observational scales. The process did not commence with observation of patient behavior, but from scrutiny of existing instruments. The underlying premise is that existing scales are based on robust, peer-reviewed observational and validation work conducted by respectable experts in the field, and thus that a meta-tool can be developed by building on this documented work and extracting the most relevant parts of each existing instrument [14,27-41]. It is therefore important to emphasise that PAIC is a meta-tool due to its inherited knowledge and evidence-base from existing instruments. The quality of the meta-tool was ensured by the systematic selection of instruments from which to derive items, as described in the methodology.

As a result, all items included in the PAIC meta-tool are considered to have face validity due to their selection by the expert panel. Thus, they are included in the current draft version pending scrutiny at the next stage of empirical reliability and validity tests. No item will be excluded until this process is complete, thus maintaining the integrity of the meta-tool approach. An example of this is the ‘empty gaze’ item which, although not immediately indicative of pain, was included by the expert panel due to its presence in the DOLOPLUS-2 scale, which has good validity data. Other items are also included which may appear to have less specificity for pain, such as anxiety. These apparently non-specific items have been included following the same rationale, with fidelity to the meta-tool approach. The further refinement stages will determine the inclusion of each item in the final version of the meta-tool, based entirely on the reliability and validity testing. It is likely that some non-specific items will succeed in this next stage of refinements since pain does not always manifest in uniquely specific behaviours. It is expected that pain-specific items will enable diagnostic differentiation of pain from other negative states and that non-specific items such as anxiety will enable a complete clinical picture of a patient. This highlights the importance of validating the draft meta-tool to understand what combination of specific and non-specific items are most valuable.

**Figure 1:**
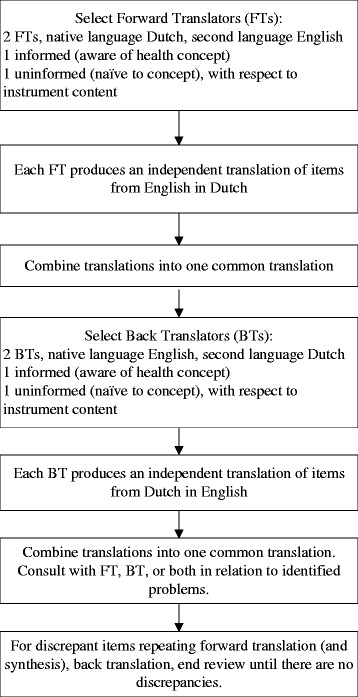
**The translation process (using English to Dutch as an example).**

The PAIC meta-tool will now be translated into at least seven European languages (German, Dutch, Romanian, Greek, Norwegian, Spanish, Italian) using the established Buffalo translation protocol [44]. Translated versions will then be checked for accuracy using the Think Aloud technique in which the translation is used in real settings by health professionals to identify errors in translation and to address any issues before use (Figure 1). The meta-tool will then be taken forward for pilot evaluation in clinical settings to establish the feasibility, reliability and clinical utility of each item using the COSMIN methodology as a criterion for psychometric testing [18]. It will also undergo additional experimental testing to determine its psychometric quality. This will be the first initiative on pain in impaired cognition to use this approach. The evaluation will enable refinement of the meta-tool to ensure the final item set will be based on the empirical, rather than theoretical performance of each item in pain assessment. These data will inform the development of a final meta-tool. Following further refinements a full clinical trial will be conducted in at least three but likely more countries across settings including hospitals, specialist secondary and primary care settings and long term care homes. This will provide a comprehensive dataset of all psychometric properties of the meta-tool and will ensure that the end result is of true utility for international use in research and clinical settings. Through the EU-COST initiative additional theoretical, qualitative and experimental evaluations will be performed to support the core assessment of the meta-tool and provide critical insight to inform the final version and its use.

The inherent strength of this work lies within the collaborative approach, which includes representatives from 16 countries. Members include aetiological, experimental and clinical (geriatric) academics, nurses, physicians, dentists, psychologists, physiotherapists and methodological experts, in addition to individuals with experience of cognitive impairment outside the field of dementia. It is the first initiative to build on the full library of existing tools, within the framework of pain assessment published by the AGS, to create a user-led assessment meta-tool for pain in dementia and cognitive impairment. We do acknowledge the potential limitations within the scope of the work, including the lack of representation from non-EU nations, particularly the US and Canada. However the EU-COST collaboration has valuable contacts in key non-member countries, such as the USA, Canada and Australia, which will be strengthened during the course of the project. To date, researchers from the US and Canada have already been consulted and involved at certain points.

There are further inherent challenges in the assessment of pain due to the subjective nature of the pain experience and lack of self-report in people with dementia. This study has utilised the best available approaches to address this issue, building on existing tools and utilising the best observational items for pain assessment to create the PAIC meta-tool. There is also a key issue regarding the established overlap between pain-related and non-pain-related behaviours, as discussed above, which blur the lines between pain assessment and other tools for use with people with dementia. However, this has been clearly recognised. In addition to the potential inclusion of non-pain specific items in the final version of the PAIC, the next phase of the initiative will involve the development of a supporting toolkit to address the issue.

The decision to create a comprehensive toolkit to support the new meta-tool is an important aspect of this work. This will provide specific resources to enable assessment of common symptoms of dementia which frequently overlap with pain-related items. These will particularly focus on BPSD such as agitation and aggression, as well as broader measures of function, activities of daily living and mood. This format will enable users to supplement the core pain assessment tool and maximise the usefulness of the overall toolkit. It is also expected that the toolkit will be tailored for use in different settings and patient groups such as Huntington’s disease, Parkinson’s disease and people with learning difficulties or delirium, according to the unique requirements and challenges these present. This may include different assessment items or specific scoring methods. The toolkit will also recognise that while self-report is usually absent in this patient group, there are cases where it is possible, for example in early stages of cognitive impairment. The toolkit will therefore include simple forms of self-report assessment for use where it is feasible. Importantly, we hope that a brief version of the toolkit for use in clinical practice will be created and fully evaluated alongside the full research tool to ensure that it will be used in everyday clinical practice. Critical stages of future work will therefore be in the effective and user-friendly design of the final toolkit to ensure its ease of use and suitability in each setting. Key additions will include considering how to identify the location and duration (acute vs chronic) of pain, the development of tailored scales for specific types of pain such as orofacial pain or back pain and the further consideration of different types and causes of pain. As a first step, the Orofacial Pain Scale for Non-Verbal Individuals (OPS-NVI) has been developed, based on recent pilot work [45]. The OPS-NVI includes observations of not only facial expressions, verbalisations & vocalisations, and body movements, but also of specific orofacial pain-related behaviours such as limited jaw movements and drooling. Observations are being made at rest, while drinking and eating, and during oral care. The instrument is currently at the stage of field testing. Another addition to the toolkit will include an instrument for the effective assessment of back pain and neuropathic pain, the latter is notoriously complex and challenging to detect in people with cognitive impairment. This issue is of high clinical relevance and is not well described in the literature, partly due to the unique assessment and treatment approaches it requires.

Finally, a dedicated dissemination Working Group will be essential to oversee an extensive strategy to ensure the implementation of the final toolkit on an international basis. This will focus on overcoming the practical, political and social obstacles to implementation, incorporating robust translation, communication and cross-working with policy makers and healthcare organisations and extensive academic, professional and lay dissemination activities.

## Conclusions

This EU-COST initiative has employed a previously unprecedented methodological approach, identifying the most meaningful pain assessment items based on empirical, rather than theoretical, value, and developing a robust evaluation and consensus procedure to create a final tool and toolkit with true universal utility. This paper represents the first step in this large, multidisciplinary, cross-cultural initiative to make tangible improvements to the management of pain in people with dementia across the EU and further afield.

## Additional file

Additional file 1:
**Table of items from the most promising pain assessment instruments categorized in Domains 1 – 3 of the AGS criteria.**

## Abbreviations

PAIC: Pain assessment in impaired cognition
AD: Alzheimer’s disease
VaD: Vascular dementia
AGS: American geriatric society
BPSD: Behavioural and psychological symptoms of dementia
OPS-NVI: Orofacial pain scale for non-verbal individuals
