# Supplementary material for: An international road map to improve pain assessment in people with impaired cognition: the development of the Pain Assessment in Impaired Cognition (PAIC) meta-tool
Source: BMC Neurol. 2014 Dec 10;14:229. doi: 10.1186/s12883-014-0229-5 (PMC4279897; doi:10.1186/s12883-014-0229-5)
Supplement: Additional file 1: — Table of items from the most promising pain assessment instruments categorized in Domains 1 – 3 of the AGS criteria. [file 12883_2014_229_MOESM1_ESM.docx]

**Supplementary Table 1: Items from the most promising pain assessment instruments categorized in Domains 1 – 3 of the AGS criteria**

| **Tool** | **AGS domain 1**  **Facial expression** | **AGS domain 2**  **Vocalization** | **AGS domain 3**  **Body movements** |
| --- | --- | --- | --- |
| **ABBEY**  (Abbey 2004) | Looking tense, frowning, grimacing, looking frightened | Whipering, groaning, crying | Fidgeting, rocking, guarding part of body, withdrawal |
| **ADD**  (Kovach 2001) | Grimacing, frowning, blinking, tightly closed or widely open eyes, frightened, weepy, worried, sad | Moaning, mumbling, chanting, grunting, whining, calling out  Screaming, crying, verbally aggressive | Tense, wringing hands, clenched fists, restless, rubbing/holding body part  hyper- or hypoactive, wandering, guarding body part, noisy breathing, resistance to care |
| **CNPI**  (Feldt 2000) | Furrowed brow, narrowed eyes, clenched teeth, tightened lips, jaw drop, distorted expressions | Sighs, gasps, moans, groans, cries, words ("ouch," "that hurts") cursing during movement; ["stop," "that's enough") | Bracing (clutching or holding onto furniture, equipment, or affected area during movement), rubbing, massaging affected area, restless, rocking |
| **DS-DAT**  (Hurley et al., 1992) | Sad: trouble looking face, looking hurt, worried, lost or lonesome, distressed appearance, sunken, “hang dog”, lackluster eyes, tears, crying; Frightened: scared, concerned, bothered fearful or troubled, alarmed experience with open eyes and pleading face; Frown: looks strained, stern or scowling looks, displeased, wrinkled brow, creases in forehead, corners of mouth turned down | Noise or speech with negative or disapproving quality, hushed low sounds such as constant muttering with a guttural tone, monotone, subdued, or varying pitched noise with a definite unpleasant sound, moaning, groaning, repeating the same words, expressing hurt or pain | Tens: extremities show tension, wringing hands, clenched fist, or knees pulled up tightly, being in a strained and inflexible position  Fidgeting: restless impatient motion, acts squirming or jittery, appearance of trying to get away from hurt area, forceful touching, tugging, or rubbing body parts |
| **DOLOPLUS 2**  (Lefebvre-Chapiro 2001) | Grimaces, Drawn, atonic fixed gaze  empty gaze, absent, tears | Words, moans, cries, voiceless, complaints | Gesture, protective actions against any investigation or nursing, protective actions taken at rest, even when not approached, restlessness |
| **EPCA-2**  (Morello 2007) | Anxious look on the face, grimaces from time to time, frightened look, tense facial expression, totally rigid expression | Complains when caregiver is present, moans or cries silently, spontaneously, shouts or complains violently and/or spontaneously | Watchful expression, seems to fear both movement and caregiver intervention, holds back or guides the hands of the caregiver, adopts a comfortable position, shows resistance when being moved or during caregiver intervention, restlessness |
| **MOBID-2**  (Husebo 2010) | Grimacing, frowning, tightening mouth, closing eyes | Ouch! Groaning, gasping, screaming | Freezing, guarding, pushing, crouching |
| **NOPPAIN**  (Snow 2004) | Grimaces, furrowed brow, winces, feared , worried | “That hurts!”, “Ouch!”, cursing, “Stop that!” moans, groans, grunts, cries, gasps, sighs | Rigidity, holding, guarding (especially during movement), massaging affected areas, restlessness, rocking |
|  |  |  |  |
| **PACSLAC**  (Fuchs-Lacelle 2004) | Grimacing, sad look, tighter face, dirty look, change in eyes (squinting, dull, bright, increased movement), frowning, pain expression, grim face, clenching teeth, wincing, opening mouth, creasing forehead, screwing up nose, teary eyed, pale face | Verbal aggression Screaming/Yelling, grunting, calling out (i.e. for help) crying, specific sound or vocalization for pain ‘ow’, ouch’, moaning and groaning, mumbling | Fidgeting, pulling away, flinching, restless, pacing, wandering, trying to leave, refusing to move, thrashing, decreased activity, refusing medications, moving slow, repetitive movements, uncooperative, resistant to care, guarding sore area, touching/holding sore area, limping, clenched fist, going into fetal position, stiff, rigid |
| **PAINAD**  (Warden 2003) | Sad (unhappy, lonesome, sorrowful, dejected look, tears in the eyes); frightened (look of fear, alarm, heightened anxiety, eyes wide open); frown(downward corners of the mouth, wrinkling in the forehead and around the mouth), grimacing (distorted, distressed look, brow is more wrinkled as is the area around the mouth, eyes may be squeezed shut) | Occasional moan or groan (mournful and murmuring sounds, wails or laments, inarticulate involuntary sounds); low level speech with a negative or disapproving quality (muttering, mumbling, whining, grumbling, swearing, complaining, sarcastic or caustic tone), repeated troubled calling out (phrases/words used over and over in a tone that suggests anxiety, uneasiness, distress); loud moaning or groaning (mournful or murmuring sounds, wails or laments louder than usual, often abruptly beginning and ending); crying (utterance of emotion accompanied by tears, sobbing and quiet weeping) | Tense (strained, apprehensive or worried, jaw may be clenched)  Distressed pacing (unsettled activities, fearful, worried or disturbed)  Fidgeting (restless movement, squirming or wiggling, hitching a chair across the room, repetitive touching, tugging, rubbing body parts)  Rigid (stiffening of the body, arms and legs are tight and inflexible, straight and unyielding trunk); fists clenched (tightly closed hands, opened and closed repeatedly); knees pulled up (flexing legs, drawing knees toward chest, troubled appearance); pulling or pushing away (resistiveness to care, trying to escape by yanking or wrenching, shoving away)  Striking out (hitting, kicking, grabbing, punching, biting) |
| **PADE**  (Villanueva 2003) | Sad, anxious, frightened, frowning, grimace, brace | Groaning, moaning, vocalization, sounds distress, gasping | Tense body language, restlessness, guard effective areas, breathing loud, breathing rapidly, hyperventilation |
| **PAINE**  (Cohen-Mansfield 2006) | Showing tension in the face, frowning, tightening the eyes, squinting, clenching or grinding teeth | Moaning, grunting, groaning, gasping, sighing, crying, whimpering, whining, screaming, yelling | Rigidity/resistance to movements: Cringing, body stiffens when touched Avoiding touch or use of affected area, pulling you toward self (toward the resident); Guarding: Abnormally stiff, interrupted, rigid movement, limping, dragging foot, impaired walking; Bracing: Leaning on wall, chair to maintain stability; moodiness, irritation, or depressed mood, seems sad, angry;  Posturing: Strange, awkward standing or sitting positions, weird movements, restlessness |
